# Supplementary material for: Machine Learning-Based Single Cell and Integrative Analysis Reveals That Baseline mDC Predisposition Correlates With Hepatitis B Vaccine Antibody Response
Source: Front Immunol. 2021 Oct 29;12:690470. doi: 10.3389/fimmu.2021.690470 (PMC8588842; doi:10.3389/fimmu.2021.690470)
Supplement: Supplementary file 1 [file DataSheet_1.docx]

**Supplemental Figures and Tables**

**Supplementary Figure 1. scRNAseq workflow -** A small cohort of human participants were challenges with the licensed Hepatitis B vaccine (*Engerix-B)*. Blood samples were collected before (Day 0) and 1, 3, 7 and 14 days after vaccination for single cell transcriptomics analysis of four different sorted innate immune cell subsets (NK cells, monocytes, mDC, and pDC) from two HBV dose 1 responders, three HBV dose 1 non-responders, and one HBV dose 1 marginal responder. Single cell RNA sequencing (scRNAseq) using a modified SmartSeq2 protocol was used for transcriptomics analysis.

**Supplementary Figure 2. Single cell isolation procedure –** A) Whole blood cell preparation procedure. B) Gating strategy for Neutrophils. C) Gating strategy for CD14^+^ HLADR^+^ monocytes, CD3^-^ CD56^+^ NK cells, and CD14^-^ HLADR^+^ dendritic cells (CD11C^+^ mDCs and CD123^high^ pDCs).

**Supplementary Figure 3. Gating strategies for mDC2 and mDC4 myeloid dendritic cells sorting** – A) Primary gating/sorting strategy. The stability of the flow cytometry run was first checked by plotting the time versus scatter plot. Areas where there was poor flow were excluded and the “good flow gate” was used to identify the cell populations of interest. Stained PBMCs were first gated for singlets (FSC-H vs. FSCA) and then gated according to their size and granularity (SSC-A vs. FSC-A). The size gate was further analysed for their uptakes of the live/dead fixable viability dye and to exclude cells expressing CD19 and CD235 surface markers. Live CD19^-^ CD235^-^ cells were further separated using CD3 surface marker to exclude T cells. The negative CD3 cells gate was carried over to identify CD14^+/-^ HLADR^+^ to further identify myeloid dendritic cells (mDC: CD11c^+^ CD123^+/-^). Finally, mDC cells were divided upon their CD1C expression to identify and sort two main mDC populations; mDC2 cells (CD1C^+^ FCER1A^+^) and mDC4 (CD1C^-^FCGR3A^+^). B) An alternative gating strategy for mDC2 and mDC4 myeloid dendritic cell subsets from mononuclear cells with CD14 exclusion. Both strategies yielded similar result; the top method was applied in this study.

**Supplementary Figure 4. FMO controls for HLADR, CD14, CD123 and CD11c -** Gating for monocytes, HLA-DR+ DCs and DC- cells using the whole staining panel or panels minus the MHCII (HLADR) or CD14 staining reagents is shown (top row). Gating for mDCs, pDCs and B cells using the whole staining panel or panels minus the CD123 or CD11c staining reagents is shown (bottom row).

**Supplementary Figure 5. NDRG2 and CDKN1C gene expression in response to polyI:C at baseline differs between HBV vaccine dose 1 responders and non-responders -** Whole blood collected from study participants prior to vaccination were incubated in Truculture tubes with or without polyI:C for 22hr at 37°C. At the end of the incubation time, plasma depleted blood was mixed with 1ml of trizol and total RNA extracted. Quantitative PCR was performed targeting NDRG2 and CDKN1C for all study participants shown in the figure. Data were normalised to the housekeeping gene (ACTB) and then to the unstimulated condition for each participant. Fold change of NDRG2 and CDKN1C expression normalized to ACTB expression (Delta Delta Ct) for each participant were determined and the data represented as ratio of the fold changes. Each colored bar shows the result of one study participant.

**Supplementary Table 1. Antibody panel used for cell sorting prior to single cell RNA sequencing**

**
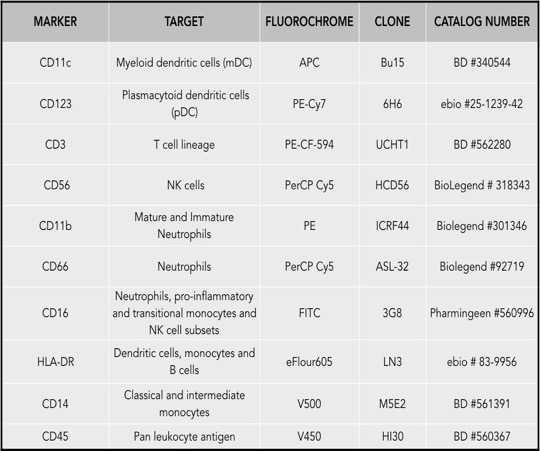
**

**Supplemental Table 2. NS-Forest v2.0 marker genes for the seven Louvain clusters** - The celltype column gives the annotated name as described in the text. The f-measure column gives the discriminatory power of the markers as a set. The True Negative, False Positive, False Negative, and True Positive gives the confusion matrix results for single cell classification using the set of markers. The markerCount column gives the total number of markers in the set followed by the symbol identities of the gene expression markers.

**Supplementary Table 3. DC2 and DC4 myeloid dendritic cells sorting panel** - Organization of panels for myeloid dendritic cells sorting from PBMC collected from peripheral whole blood. Nine-color panels were established in order to specifically sort mDC2 and mDC4 subsets as described in Supplementary Figure 3.

**Supplementary Table 4. CD4 and CD8 T cells proliferation staining panel** - Organization of panels for assessing autologous T cell proliferation in presence of either mDC4 or mDC2 dendritic cells. Cell proliferation was assessed by Oregon Green. The eight-color panel was established in order to quantify CD4 and CD8 proliferation after 5 days.

**Supplemental Table 5. qRT-PCR results –** 96 mDCs were sorted into wells of a microtiter plate, cDNA prepared and qPCR for ACTB, CDKN1C and NDRG2 performed as described in the Methods section. The number of cells positive by qPCR is shown in the “count” columns. The percent of ACTB+ cells that were also positive for indicated marker gene is shown in the “%” columns. The relative proportion of NDRG2-expressing cells/CDKN1C-expressing cells is shown in the last column. Data for participant GR01 is shown as an example.

**Supplemental Table 6. Relative proportion of NDRG2-expressing cells/CDKN1C-expressing cells** – Relative proportion of NDRG2-expressing cells/CDKN1C-expressing cells were calculated as described in Supplemental Table 4 for all samples and all participant. Numbers in grey were interpolated from flanking timepoints because no cells were detected for at least one of the two mDC subsets in these samples.
